# Associations between self-reported diet during treatment and chemotherapy-induced peripheral neuropathy in a cooperative group trial (S0221)

**DOI:** 10.1186/s13058-018-1077-9

**Published:** 2018-11-28

**Authors:** Jennifer M. Mongiovi, Gary R. Zirpoli, Rikki Cannioto, Lara E. Sucheston-Campbell, Dawn L. Hershman, Joseph M. Unger, Halle C. F. Moore, James A. Stewart, Claudine Isaacs, Timothy J. Hobday, Muhammad Salim, Gabriel N. Hortobagyi, Julie R. Gralow, G. Thomas Budd, Kathy S. Albain, Christine B. Ambrosone, Susan E. McCann

**Affiliations:** 1 University at Buffalo, Buffalo, NY USA; 2 Roswell Park Cancer Institute, Buffalo, NY USA; 3 Boston University, Boston, MA USA; 4 The Ohio State University, Columbus, OH USA; 5 Columbia University, New York, NY USA; 6 SWOG Statistical Center, Fred Hutchinson Cancer Research Center, Seattle, WA USA; 7 Cleveland Clinic, Cleveland, OH USA; 8 Baystate Medical Center, Springfield, MA USA; 9 Georgetown University, Washington, DC USA; 10 Mayo Clinic, Rochester, MN USA; 11 Allan Blair Cancer Centre, Regina, SK Canada; 12 The University of Texas MD Anderson Cancer Center, Houston, TX USA; 13 Seattle Cancer Care Alliance, Seattle, WA USA; 14 Loyola University Chicago Stritch School of Medicine, Chicago, IL USA; 15 Cancer Prevention and Control, Roswell Park Cancer Institute, Elm and Carlton Streets, Buffalo, NY 14263 USA

**Keywords:** Chemotherapy-induced peripheral neuropathy, Breast cancer, Diet, Taxane, Peripheral nervous system diseases

## Abstract

**Background:**

The pathophysiology of chemotherapy-induced peripheral neuropathy (CIPN) is not well understood. Currently, dose reduction is the only recommendation for alleviating symptoms, often leading to premature treatment cessation. The primary aim of this analysis was to determine the association between components of diet during taxane treatment for breast cancer and change in CIPN symptoms over treatment.

**Methods:**

Women with stage II or III invasive breast cancer were enrolled into an ancillary study to the North American Breast Cancer Intergroup phase III trial (S0221) led by the Southwest Oncology Group (SWOG). Questionnaires including a food frequency questionnaire and the Functional Assessment of Cancer Treatment Gynecologic Oncology Group—Neurotoxicity were administered to assess diet and neuropathic conditions at baseline and during chemotherapy. Ordinal regression was used to estimate odds ratios (ORs) for associations between various food groups and change in neuropathy score (< 10%, 10–30%, > 30%) (*n* = 900).

**Results:**

The odds of worse neuropathy decreased by 21% for each increase in tertile of grain consumption (OR = 0.79, 95% CI 0.66–0.94, *p* = 0.009). We also observed a nominal 19% increase with higher consumption of citrus fruits (OR = 1.19, 95% CI 1.01–1.40, *p* = 0.05).

**Conclusions:**

Distinguishing between those who experienced a moderate and a severe change in neuropathy, we found that citrus fruit and grain consumption may play a role in the severity of symptoms. Since there are no existing dietary recommendations for the management of CIPN, further research is needed to investigate whether there may be certain foods that could worsen or alleviate neuropathy symptoms associated with treatment for breast cancer.

**Trial registration:**

ClinicalTrials.gov, NCT03413761. Registered retrospectively on 29 January 2018.

## Background

Although effective for cancer treatment, use of chemotherapy drugs often results in intolerable side effects that may deter patients from completing planned treatments. One of the most common reasons for prematurely discontinuing treatment with drugs such as taxanes, epothilones, and vinca alkaloids is the development of chemotherapy-induced peripheral neuropathy (CIPN) [1, 2]. The most common CIPN symptoms include burning, tingling, numbness, loss of proprioceptive sense, increased sensitivity to pain, and reduced reflexes in the hands and feet. Aside from discomfort, CIPN symptoms can cause loss of functional ability, affecting a patient’s ability to perform daily activities and increasing the prevalence of falls [2, 3].

While the exact prevalence of CIPN is not known, a recent meta-analysis estimated that approximately 50% of breast cancer patients develop CIPN during taxane treatment, with up to 80% of those still experiencing symptoms at 6 months post treatment [4, 5]. While some women experience improvement, over 40% have reported symptoms at 3 years or more following treatment [6–8]. The specific pathophysiology of CIPN is not well understood, leaving patients with little to no options to prevent these potentially debilitating side effects [9]. As a result, dose reduction can be recommended for alleviating acute symptoms and frequently leads to a premature cessation of treatment for those with severe neuropathy [4].

Despite the importance of this clinical issue, the American Society for Clinical Oncology treatment and prevention practice guidelines concluded that insufficient evidence exists to support use of many nonpharmaceutical interventions and that additional research is warranted [5]. Some emerging literature suggests that lifestyle factors including body mass index (BMI), physical activity, diet, and dietary supplement use may play a role in the development and severity of CIPN [10]. Recently, we reported that multivitamin use prior to and during treatment was associated with reduced symptoms of CIPN among breast cancer patients in the Diet, Exercise, Lifestyle, and Cancer Prognosis (DELCaP) study [11]. Many individuals, especially breast cancer patients, may use dietary supplements to increase vitamin and mineral consumption as a complement to traditional cancer therapies [12, 13].

Dietary modifications have been explored in an effort to self-manage neuropathy symptoms. A vegetarian diet specifically has been found to improve symptoms among those with diabetic neuropathy. It is unclear whether this is due to the increased consumption of plant-based foods containing a higher density of vitamins or avoidance of certain foods [14, 15]. The association between modifiable factors and CIPN is an understudied area. To better understand the relationship between diet and CIPN, we examined food groups consumed during chemotherapy treatment and development of neuropathy symptoms to determine whether an association exists between diet and neuropathy severity.

## Methods

### Participants

Data were obtained from the DELCaP study, an ancillary study to a phase III therapeutic trial (S0221) led by the South West Oncology Group (SWOG) (ClinicalTrials.gov NCT00070564). DELCaP was developed to assess diet and lifestyle data at multiple time points including baseline, during, and post treatment. Detailed recruitment procedures, randomization procedures, and inclusion criteria for participation in both S0221 and DELCaP have been described previously [11, 16]. Briefly, women enrolled in S0221 had a confirmed diagnosis of stage II or III invasive breast cancer and were randomized to one of four treatment arms. Each patient received either doxorubicin plus cyclophosphamide every 2 weeks with pegfilgrastim or weekly doxorubicin plus daily cyclophosphamide with filgrastim. Patients then either received 12 weekly cycles of paclitaxel or paclitaxel every 2 weeks with pegfilgrastim for six cycles. Upon enrollment into S0221, participants were contacted for participation in the DELCaP study. Specifically, informed consent was obtained during the consent for S0221, allowing research staff to contact potential participants for inclusion in the DELCaP study. This study was approved by the Institutional Review Board at Roswell Park Cancer Institute and all participating institutions that enrolled patients.

A total of 1468 participants participated in the DELCaP study; 1460 participants completed the baseline questionnaire including foods usually eaten over the last 12 months prior to cancer diagnosis, and 1234 patients completed the 6-month follow-up regarding diet during treatment. In order to allow for the consideration of repeated measures, 226 participants who did not provide information on at least 50% of foods in both the baseline and follow-up questionnaires or had a difference > 10 in the number of foods missing between baseline and follow-up were excluded from this analysis. An additional 340 participants who did not complete or had missing values for the baseline or follow-up Functional Assessment of Cancer Treatment Gynecologic Oncology Group—Neurotoxicity (FACT/GOG-Ntx) questionnaire were not included. The total study sample included 900 women in this analysis.

### Data collection

DELCaP questionnaires were administered at four time points throughout the study: at baseline, following completion of treatment, and annually for the following 2 years. A baseline questionnaire (Q1), administered at study enrollment but before treatment, included questions on race and ethnicity, menopausal status, height and weight, smoking history, alcohol consumption, a 110-item food frequency questionnaire (FFQ) including detailed questions regarding vitamin and dietary supplement use, and the FACT/GOG-Ntx scale to assess neuropathic conditions. The FFQ was adapted from the validated Vitamins and Lifestyle (VitaL) study [17]. A second questionnaire (Q2) was mailed to participants 6 months after registration to the trial, when chemotherapy should have been completed. For the purpose of the current analyses, we compared baseline to Q2 neuropathy symptoms to determine the change in neuropathy symptoms resulting from chemotherapy treatments and FFQ data from Q2 to determine diet during treatment (6-month recall).

For quality control purposes, data entry was performed twice by different research staff and compared for accuracy and resolution of discrepancies.

### Exposure assessment

In the baseline questionnaire, participants were asked to indicate how often each food and beverage was usually consumed over the last 12 months prior to diagnosis. Food consumption frequency included never, 1 per month, 2–3 per month, 1 per week, 2 per week, 3–4 per week, 5–6 per week, 1 per day, and 2+ per day, with additional options for a small, medium, and large serving size for all foods. Beverage consumption frequency included never, < 1 per month, 1–3 per month, 1 per week, 2–4 per week, 5–6 per week, 1 per day, 2–3 per day, 4–5 per day, and 6+ per day with small, medium, and large serving size options. Standard medium serving sizes were provided for reference for both food and beverages. Missing food frequency values among those not missing > 50% of foods were treated as a value of 0 (not eaten). Missing serving sizes were assigned a default medium value. An aggregate monthly total for each food was computed by converting small, medium, and large serving sizes into values of 0.5, 1, and 1.5, respectively, which was then multiplied by the total monthly frequency. The monthly sums of specific foods were totaled to create food groups based on the University of Minnesota Nutrition Data System for Research and MyPlate food groups (Table 1) [18, 19]. Food groups included citrus fruits, other fruits, dark green vegetables, red/orange vegetables, starchy vegetables, cruciferous vegetables, beans/bean dishes, other vegetables, fish, poultry, red meat, processed meat, other protein, dairy, grains, sweets, fried foods, added fats, and alcohol. The follow-up questionnaire included the same FFQ and asked participants to indicate their diet over the previous 6 months (presumably reflecting diet during treatment) during the time period in which neuropathy symptoms would have developed or worsened. Data from this FFQ were used in analysis and modeled as ordinal variables.

**Table 1:** Classification of foods and food groups reported by patients enrolled in S0221^a^

Variable	FFQ items included	Individual foods included
All fruits	Citrus fruits, other fruits	(See listing below)
Citrus fruits	Oranges, orange juice	Oranges, grapefruit, and tangerines; 100% orange juice and grapefruit juice
Other fruits	Apples, bananas, peaches, apricots, dried fruit, berries, melons, other fruits, 100% fruit juice	Apples, apple sauce, and pears; bananas; peaches, nectarines, and plums; apricots (fresh or canned); dried fruit (other than apricots) such as raisins or prunes; berries such as strawberries and blueberries; cantaloupe, other melons, and mango; any other fruit such as fruit cocktail, pineapples, and cherries; other 100% fruit juice
All vegetables	Dark green, red/orange, starchy, cruciferous, beans/bean dishes, other vegetables	(See listing below)
Dark green	Salad, greens	Green salad (lettuce or spinach); cooked greens such as spinach, mustard greens, or collards
Red/orange	Salsa, tomatoes, carrots, winter squash, tomato juice	Salsa (as in dip or foods); fresh tomatoes; carrots; winter squash such as acorn or butternut, sweet potatoes and yams; tomato juice, V-8, and other vegetable juice
Starchy	Peas, boiled potatoes, corn	Green peas; potatoes (boiled, baked, or mashed); corn
Cruciferous	Broccoli, cauliflower	Broccoli; cauliflower, cabbage, and Brussels sprouts
Beans/bean dishes	Beans, bean soups	Beans such as baked, refried, and chili without meat; bean soups such as pea, lentil, and black bean
Other vegetables	Peppers, green beans, summer squash, onions, garlic, avocado	Green and red peppers and chilies; green or string beans; summer squash, zucchini, and okra; onions and leeks; fresh garlic including in cooking; avocado and guacamole
Protein sources		
Fish	Tuna, shell fish, white fish, dark fish	Canned tuna, tuna salad, tuna casserole; shellfish, not fried (shrimp, lobster, crab, and oysters; white fish (broiled or baked) such as sole, halibut, and cod; dark fish (broiled or baked) such as tuna or salmon
Poultry	Chicken liver, roasted chicken	Liver, chicken liver, and organ meats; chicken and turkey (roasted, stewed, or broiled)
Red meat	Beef, ground meat	Beef, pork, ham, and lamb; ground meat including hamburgers and meatloaf
Processed meat	Bacon, low or reduced fat hot dog, regular hot dog, lunch meats, other lunch meats	Bacon and breakfast sausage; low or reduced fat hot dogs and sausage; lunch meats such as ham, turkey, and low fat bologna; all other lunch meat such as bologna, salami, and Spam
Other protein	Peanut butter, tofu, eggs	Peanut butter, peanuts, and other nuts and seeds; tofu, tempeh, and products such as tofu hot dogs, soy burgers, and tofu cheese; eggs
Other foods		
Dairy	Cottage and ricotta cheese, low or reduced fat cheese, all other cheese, yogurt, milk, soymilk, rice milk, milk added to cereal	Cottage cheese and ricotta cheese; low or reduced fat cheese, including cheese used in cooking; all other cheeses, such as American, cheddar, or cream cheese, including cheese used in cooking; yogurt, all types, except for frozen; milk as a beverage; soy milk; rice milk
Grains	Cold cereal, cooked cereal, pancakes, muffins, white bread, granola bar, sports or meal replacement bar, low or nonfat chips, regular chips, low or nonfat crackers, regular crackers, grains	Cold cereal; cooked cereals and grits; pancakes, French toast, and waffles; muffins, scones, croissants, and biscuits; white breads including bagels, rolls, and English muffins; granola bars and cereal bars such as NutriGrain Bars; sports or meal replacement bars such as Power Bars and Cliff Bars; low or nonfat potato and tortilla chips, pretzels, and plain or low fat microwave popcorn; regular potato and tortilla chips, puffs, and microwave or buttered popcorn; low or nonfat crackers such as saltines and SnackWell’s; regular crackers such as Ritz and Wheat Thins; rice noodles and other grains (as side dish)
Sweets	Jam, donuts, ice cream, frozen yogurt, pudding, cookies and cakes, chocolate and candy, cranberry juice, fruit drinks, soft drinks	Jam, jelly, honey, and syrup; ice cream and milk shakes; low or nonfat frozen desserts such as low fat ice cream, frozen yogurt, and sherbet; pudding, custard, and flan; donuts, pies, and pastries; cookies and cakes; chocolate, candy bars, and toffee; cranberry juice and other fruit juice cocktails; fruit drinks fortified with vitamin C, such as Hi-C; regular soft drinks
Fried foods	Fried potatoes, fried fish, fried chicken	French fries, fried potatoes, and hash browns; fried fish, fish sandwich and fried shellfish (shrimp, oysters); fried chicken including chicken nuggets and tenders
Added fats	Butter on bread, butter added to dishes, mayonnaise, salad dressing, gravy	Butter or margarine on breads, hot cereals, pancakes, etc.; butter, margarine, sour cream, and other fat added to vegetables, potatoes, and rice; mayonnaise and mayonnaise-type spreads; salad dressing (all types); meat gravies
Alcohol	Beer, red wine, white wine, liquor	Beer (all types); red wine; white or rose wine; liquor and mixed drinks

*FFQ* food frequency questionnaire

^a^Based on the University of Minnesota Nutrition Data System for Research and MyPlate food group classification [18, 19]

### Outcome assessment

At baseline and in the follow-up questionnaire, participants completed an 11-item FACT/GOG-Ntx scale to assess the severity of neuropathy symptoms during the previous 7 days. Symptoms including numbness in hands and feet, discomfort in hands and feet, joint and muscle pain, hearing and ear trouble, trouble feeling, and trouble walking were assessed on a 5-point scale ranging from 4 = “Not at all”, 3 = “A little bit”, 2 = “Somewhat”, 1 = “Quite a bit”, to 0 = “Very much”. Total baseline and 6-month follow-up scores were computed and used to determine the percentage decrease in neuropathy score, indicating a worsening of symptoms. Previous literature has determined that a 10% or greater decrease in FACT/GOG-Ntx score is clinically meaningful for assessment of neuropathy [10, 20, 21]. Scores were grouped into approximate tertiles based on whether the participant experienced no to minimal increase (< 10%), moderate increase (10–30%), or severe increase (> 30%) in severity of neuropathy symptoms and were modeled as an ordinal variable.

### Statistical analysis

Demographic characteristics obtained from baseline questionnaires included age, race, height, weight, menopausal status, smoking status, highest education obtained, and marital status. Height and weight at baseline and follow-up were used to determine the BMI and whether the participant had changed weight during treatment. A chi-square test for independence and one-way analysis of variance were performed to test for differences in neuropathy score frequencies across groups. Food groups were categorized as tertiles of monthly servings during chemotherapy treatment and compared using Pearson’s chi-square. Food frequencies at baseline and follow-up were not independent and were predictive of the other time point (0.36 ≤ *r* ≤ 0.58), which is commonly observed in other studies of long-term reproducibility and considered to fall within an acceptable range for FFQs [22, 23]. Therefore, baseline FFQ data were not included in the analysis.

Ordinal regression was used to estimate odds ratios (ORs) and 95% confidence intervals (95% CIs) for the associations between each food group and change in neuropathy score. This modeling approach takes into account the natural order of the multiple category outcome, change in reported neuropathy score, or increasing severity. The ordinal model also assumed that the odds ratio of each outcome category was independent of other categories (proportional odds assumption) [24]. The proportional odds assumption was first tested for associations between the outcome and each food group as well as the final model. Study arm, age in years, self-identified race or ethnicity (non-Hispanic White, Spanish/Latino/Hispanic, Black/African American, other), BMI calculated from self-reported height and weight, change in weight from diagnosis to post treatment (lost weight, maintained weight, gained weight), menopausal status (pre, post), smoking status (never, former, current), highest level of education, and marital status were assessed as potential covariates. Details regarding randomization of participants and the dose and schedule of treatment have been described previously [16]. Variables that were significantly associated with both neuropathy category and at least two food groups at *p* ≤ 0.20 were included in the final adjusted model (age, race, BMI at baseline, and smoking status). The final adjusted model included a single OR for CIPN severity associated with each food group meeting the proportional odds assumption and was interpreted as the odds of reporting worse neuropathy at follow-up for increased consumption of each food group (in tertiles) [24]. Statistical tests performed were two-sided at α = 0.05. All analyses were performed using SAS 9.4 (SAS Institute, Cary, NC, USA).

## Results

As presented in Table 2, the mean age at baseline was 52.0 (SD = 9.7) years and the majority of women identified as non-Hispanic White (84.7%). As suggested by the relatively young mean age, only 54.8% of women were postmenopausal. Most were overweight or obese prior to treatment (67.9%), with slightly fewer obese participants at follow-up (33.1% vs 35.6%); only 6.2% experienced a greater than 10% decrease in body weight. The majority of women reported never having smoked (57.0%), at least some college or technical school (73.4%), and being married or living as married (76.2%). Women who experienced worse neuropathy were statistically significantly older (*p* = 0.003), overweight or obese (*p* = 0.03), experienced a change in weight from baseline (*p* = 0.007), and were postmenopausal (*p* = 0.009). Most women experienced either a moderate (34.0%) or severe (34.8%) increase in neuropathy symptoms at the completion of treatment.

**Table 2:** Change in self-reported neurotoxicity scores (FACT/GOG-Ntx subscale) pre to post chemotherapy treatment by population characteristics (*n* = 900)

	Total	< 10%^a^	10–30%^a^	> 30%^a^	*p* value^b^
Characteristic (mean ± SD)		281 (31.2)	306 (34.0)	313 (34.8)	
Age at baseline (years) (52.0 ± 9.7)^c^					0.003
< 40	93 (10.3)	33 (11.7)	43 (14.1)	17 (5.4)	
40–49	275 (30.6)	97 (34.5)	87 (28.5)	91 (29.1)	
50–59	323 (35.9)	92 (32.7)	109 (35.7)	122 (39.0)	
≥ 60	208 (23.1)	59 (21.0)	66 (21.6)	83 (26.5)	
Race/ethnicity					0.98
Non-Hispanic White	762 (84.7)	238 (84.7)	259 (84.6)	265 (84.7)	
Spanish/Latino/Hispanic	27 (3.0)	7 (2.5)	9 (2.9)	11 (3.5)	
Black/African American	56 (6.2)	19 (6.8)	20 (6.5)	17 (5.4)	
Other	55 (6.1)	17 (6.1)	18 (5.9)	20 (6.4)	
BMI at baseline (kg/m^2^)^c^ (28.7 ± 6.5)					0.03
Underweight (< 18.5)	8 (0.9)	6 (2.2)	1 (0.3)	1 (0.3)	
Normal/underweight (18.5–24.9)	278 (31.3)	103 (37.1)	103 (34.1)	72 (23.3)	
Overweight (25.0–29.9)	287 (32.3)	84 (30.22)	85 (28.2)	118 (38.2)	
Obese (30.0+)	316 (35.6)	85 (30.6)	113 (37.4)	118 (38.2)	
BMI at follow-up (kg/m^2^)^c^ (28.5 ± 6.4)					0.16
Underweight (< 18.5)	8 (0.9)	4 (1.4)	2 (0.7)	2 (0.7)	
Normal (18.5–24.9)	281 (31.7)	103 (37.2)	99 (33.0)	79 (25.6)	
Overweight (25.0–29.9)	304 (34.3)	93 (33.6)	88 (29.3)	123 (39.8)	
Obese (30.0+)	293 (33.1)	77 (27.8)	111 (37.0)	105 (34.0)	
Change in weight during treatment					0.007
Lost weight	56 (6.2)	8 (2.9)	15 (4.9)	33 (10.5)	
Did not change	772 (85.8)	256 (91.1)	263 (86.0)	253 (80.8)	
Gained weight	72 (8.0)	17 (6.1)	28 (9.2)	27 (8.6)	
Menopausal status					0.009
Pre	407 (45.2)	135 (48.0)	152 (49.7)	120 (38.3)	
Post	493 (54.8)	146 (52.0)	154 (50.3)	193 (61.7)	
Smoking status					0.09
Never	511 (57.0)	169 (60.4)	182 (59.7)	160 (51.3)	
Former	285 (31.8)	84 (30.0)	94 (30.8)	107 (34.3)	
Current	101 (11.3)	27 (9.6)	29 (9.5)	45 (14.4)	
Highest education					0.21
Did not complete high school	51 (5.7)	22 (7.9)	11 (3.6)	18 (5.8)	
High school	188 (21.0)	54 (19.4)	63 (20.7)	71 (22.7)	
Some college or technical school	325 (36.3)	97 (34.8)	105 (34.5)	123 (39.3)	
College graduate	196 (21.9)	64 (22.9)	70 (23.0)	62 (19.8)	
Advanced degree	136 (15.2)	42 (15.1)	55 (18.1)	39 (12.5)	
Marital status					0.80
Married/living as married	682 (76.2)	216 (77.1)	232 (76.6)	234 (75.0)	
Widowed	38 (4.3)	15 (5.4)	9 (3.0)	14 (4.5)	
Divorced/separated	127 (14.2)	35 (12.5)	45 (14.9)	47 (15.1)	
Single/never married	48 (5.4)	14 (5.0)	17 (5.6)	17 (5.5)	

Data presented as *N* (%)

*SD* standard deviation, *FACT/GOG-Ntx* Functional Assessment of Cancer Treatment Gynecologic Oncology Group—Neurotoxicity

^a^Percent increase in neuropathy severity (based on self-reported score)

^b^Chi-square test for independence, analysis of variance for continuous variables (α = 0.05)

^c^Modeled as continuous variable

No significant differences in fruit and vegetable consumption were observed across categories of neuropathy (Table 3). Women with higher grain consumption had less change in neuropathy (39.2%) whereas those who had the lowest grain consumption had a greater increase in neuropathy score (40.3%, *p* = 0.01) (Table 4). Those who reported the greatest increase in neuropathy symptoms were more likely to abstain from alcohol than those who reported little to no change (63.3% vs 55.6% vs 58.0%, *p* = 0.009). A greater proportion of those who reported consuming up to two servings of alcohol per month experienced a moderate change in neuropathy scores (27.5%) than those who reported a severe change (15.7%) or no change (20.6%). Those who reported consuming over two servings of alcohol were least likely to experience a moderate change (17.0%).

**Table 3:** Self-reported change in neurotoxicity scores by tertiles of monthly servings^a^ of fruit and vegetable during treatment (FACT/GOG-Ntx subscale) (*n* = 900)

Food group	Total	< 10%^a^	10–30%^b^	> 30%^b^	*p* value ^c^
All fruits					0.81
≤ 30.8	300 (33.3)	96 (34.2)	100 (32.7)	104 (33.2)	
30.9–69.5	303 (33.7)	88 (31.3)	103 (33.7)	112 (35.8)	
> 69.5	297 (33.0)	97 (34.5)	103 (33.7)	97 (31.0)	
Citrus fruits					0.38
≤ 2.5	308 (34.2)	100 (35.6)	109 (35.6)	99 (31.6)	
2.6–14.5	311 (34.6)	101 (35.9)	94 (30.7)	116 (37.1)	
> 14.5	281 (31.2)	80 (28.5)	103 (33.7)	98 (31.3)	
Other fruits					0.74
≤ 22.8	300 (33.3)	91 (32.4)	97 (31.7)	112 (35.8)	
22.9–53.0	300 (33.3)	94 (33.5)	109 (35.6)	97 (31.0)	
> 53.0	300 (33.3)	96 (34.2)	100 (32.7)	104 (33.2)	
All vegetables					0.57
≤ 39.8	302 (33.6)	93 (33.1)	99 (32.4)	110 (35.1)	
39.9–76.0	300 (33.3)	103 (36.7)	100 (32.7)	97 (31.0)	
> 76.0	298 (33.1)	85 (30.3)	107 (35.0)	106 (33.9)	
Dark green					0.14
≤3.8	276 (30.7)	85 (30.3)	88 (28.8)	103 (32.9)	
3.9–12.0	333 (37.0)	103 (36.7)	105 (34.3)	125 (39.9)	
> 12.0	291 (32.33)	93 (33.1)	113 (36.9)	85 (27.2)	
Red/orange					0.49
≤ 8.0	316 (35.1)	97 (34.5)	99 (32.4)	120 (38.3)	
8.1–20.5	291 (32.3)	97 (34.5)	100 (32.7)	94 (30.0)	
> 20.5	293 (32.6)	87 (31.0)	107 (35.0)	99 (31.6)	
Starchy					0.36
≤ 5.3	306 (34.0)	88 (31.3)	100 (32.7)	118 (37.7)	
5.4–11.0	299 (33.2)	102 (36.3)	105 (34.3)	92 (29.4)	
> 11.0	295 (32.8)	91 (32.4)	101 (33.0)	103 (32.9)	
Cruciferous					0.79
≤ 2.5	334 (37.1)	97 (34.5)	119 (38.9)	118 (37.7)	
2.6–6.5	275 (30.6)	93 (33.1)	89 (29.1)	93 (29.7)	
> 6.5	291 (32.3)	91 (32.4)	98 (32.0)	102 (32.6)	
Beans/bean dishes					0.74
≤ 0.5	336 (37.3)	105 (37.4)	106 (34.6)	125 (39.9)	
0.6–2.5	284 (31.6)	89 (31.7)	99 (32.4)	96 (30.7)	
> 2.5	280 (31.1)	87 (31.0)	101 (33.0)	92 (29.4)	
Other vegetables					0.34
≤ 8.0	301 (33.4)	98 (34.9)	93 (30.4)	110 (35.1)	
8.1–20.5	305 (33.9)	97 (34.5)	114 (37.3)	94 (30.0)	
> 20.5	294 (32.7)	86 (30.6)	99 (32.4)	109 (34.8)	

Data presented as *N* (%)

*FACT/GOG-Ntx* Functional Assessment of Cancer Treatment Gynecologic Oncology Group—Neurotoxicity

^a^Serving size based on United States Department of Agriculture portion sizes

^b^Percent increase in neuropathy severity (based on self-reported score)

^c^Chi-square (α = 0.05)

**Table 4:** Self-reported change in neurotoxicity scores by tertiles of monthly servings^a^ of meats and other foods during treatment (FACT/GOG-Ntx subscale) (*n* = 900)

Food group	Total	< 10%^a^	10–30%^b^	> 30%^b^	*p* value^c^
Protein					
Fish					0.98
≤ 1.5	320 (35.6)	101 (35.9)	110 (36.0)	109 (34.8)	
1.6–5.0	287 (31.9)	91 (32.4)	98 (32.0)	98 (31.3)	
> 5.0	293 (32.6)	89 (31.7)	98 (32.0)	106 (33.9)	
Poultry					0.90
≤ 2.0	306 (34.0)	93 (33.1)	104 (34.0)	109 (34.8)	
2.1–4.0	296 (32.9)	89 (31.7)	105 (34.3)	102 (32.6)	
> 4.0	298 (33.1)	99 (35.2)	97 (31.7)	102 (32.6)	
Red meats					0.98
≤ 5.0	319 (35.4)	97 (34.5)	111 (36.3)	111 (35.5)	
5.1–12.0	281 (31.2)	91 (32.3)	92 (30.1)	98 (31.3)	
> 12.0	300 (33.3)	93 (33.1)	103 (33.7)	104 (33.2)	
Processed meat					0.87
≤ 3.3	306 (34.0)	95 (33.8)	102 (33.3)	109 (34.8)	
3.4–8.8	294 (32.7)	89 (31.7)	107 (35.0)	98 (31.3)	
> 8.8	300 (33.3)	97 (34.5)	97 (31.7)	106 (33.9)	
Other proteins					0.47
≤ 6.0	311 (34.6)	97 (34.5)	98 (32.0)	116 (37.1)	
6.1–15.3	296 (32.9)	85 (30.3)	107 (35.0)	104 (33.3)	
> 15.3	293 (32.6)	99 (35.2)	101 (33.0)	93 (29.7)	
Other foods					
Dairy					0.31
≤ 16.3	300 (33.3)	89 (31.7)	99 (32.4)	112 (35.8)	
16.3–39.5	300 (33.3)	87 (31.0)	113 (36.9)	100 (32.0)	
> 39.5	300 (33.3)	105 (37.4)	94 (30.7)	101 (32.3)	
Grains					0.01
≤ 27.5	301 (33.4)	78 (27.8)	97 (31.7)	126 (40.3)	
27.6–48.5	299 (33.2)	93 (33.1)	109 (35.6)	97 (31.0)	
> 48.5	300 (33.3)	110 (39.2)	100 (32.7)	90 (28.8)	
Sweets					0.31
≤ 15.5	305 (33.9)	96 (34.2)	93 (30.4)	116 (37.1)	
15.6–35.0	296 (32.9)	98 (34.9)	99 (32.4)	99 (31.6)	
> 35.0	299 (33.2)	87 (30.96)	114 (37.3)	98 (31.3)	
Fried foods					0.47
≤ 2.0	309 (34.3)	86 (30.6)	112 (36.6)	111 (35.5)	
2.1–5.3	302 (33.6)	105 (37.4)	97 (31.7)	100 (32.0)	
> 5.3	289 (32.1)	90 (32.0)	97 (31.7)	102 (32.6)	
Added fats					0.41
≤ 17.3	303 (33.7)	89 (31.7)	98 (32.0)	116 (37.1)	
17.3–34.0	299 (33.2)	103 (36.7)	101 (33.0)	95 (30.4)	
> 34.0	298 (33.1)	89 (31.7)	107 (35.0)	102 (32.6)	
Alcohol					0.009
0.0	531 (59.0)	163 (58.0)	170 (55.6)	198 (63.3)	
0.1–2.0	191 (21.2)	58 (20.6)	84 (27.5)	49 (15.7)	
> 2.0	178 (19.8)	60 (21.4)	52 (17.0)	66 (21.1)	

Data presented as *N* (%)

*FACT/GOG-Ntx* Functional Assessment of Cancer Treatment Gynecologic Oncology Group—Neurotoxicity

^a^Serving size based on United States Department of Agriculture portion sizes

^b^Percent increase in neuropathy severity (based on self-reported score)

^c^Chi-square (α = 0.05)

**Fig. 1:**
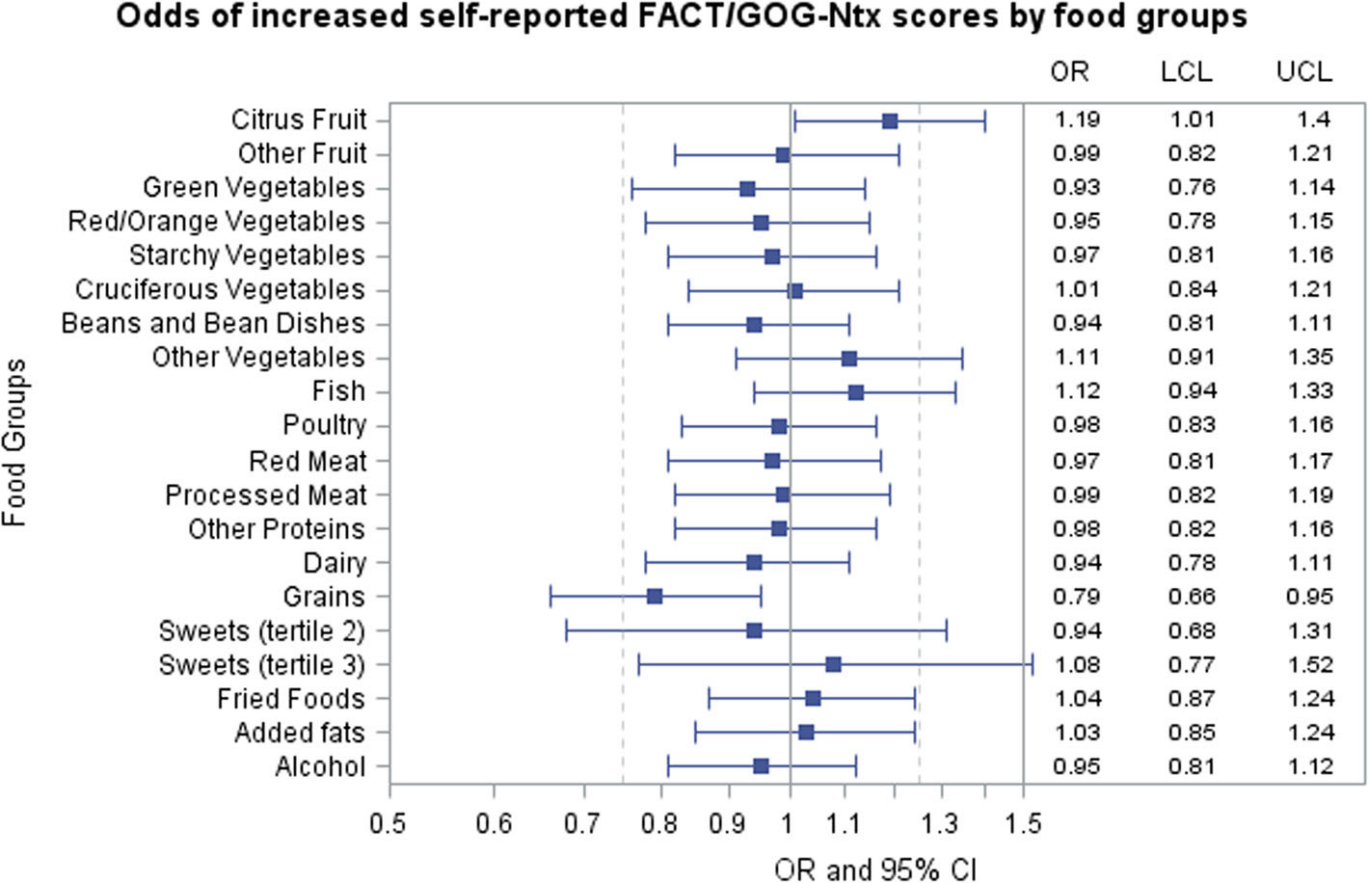
Odds of increased self-reported FACT/GOG-Ntx scores and 95% CI by food groups consumed during treatment (*n* = 900). OR and 95% CI estimated with ordinal logistic regression adjusted for age at baseline, race/ethnicity, BMI at baseline, smoking status, and menopausal status. CI confidence interval, FACT/GOG-Ntx, Functional Assessment of Cancer Treatment Gynecologic Oncology Group—Neurotoxicity, LCL lower confidence limit, OR odds ratio, UCL upper confidence limit

The proportional odds assumption held for associations between CIPN severity and all food groups except for sweets, which was then treated as a nominal categorical variable. The referent category for each food group was the lowest tertile of intake. We observed a significant unadjusted association between grains and change in neuropathy (*p* = 0.002) suggesting that higher grain consumption was associated with less of an increase in neuropathy symptoms (OR = 0.76, 95% CI 0.63–0.90) (data not shown). Both citrus fruits (OR = 1.08, 95% CI 0.93–1.25) and alcohol (OR = 0.94, 95% CI 0.80–1.09) were not significant. The final adjusted model included age at baseline, race/ethnicity, BMI at baseline, smoking status, and menopausal status as well as all food groups. In this model, the odds of reporting worse neuropathy decreased by 21% for each increase in tertile of grain consumption (OR = 0.79, 95% CI 0.66–0.94, *p* = 0.009) (Fig. 1). We also observed a 19% increase with increasing consumption of citrus fruits (OR = 1.19, 95% CI 1.01–1.40, *p* = 0.05) in the final adjusted model, although the risk estimate was not statistically significant.

The majority of women reported increasing severity of sensory neuropathy, which contributes most heavily to the overall CIPN score. When analyses were limited to the sensory component of the CIPN score, we observed associations similar to those seen with the overall score for all food groups with the exception of sweets. Odds of reporting worse sensory neuropathy increased with each additional tertile of sweet consumption (OR = 1.27, 95% CI 0.90–1.79; OR = 1.44, 95% CI 0.99–2.09, tertile 2 and 3, respectively) (data not shown).

## Discussion

In this study of diet during chemotherapy treatment in women with breast cancer, we observed that consumption of certain foods was associated with greater development of CIPN symptoms. In our study population, we observed that reporting worse neuropathy was associated with increasing age, being overweight or obese, a change in weight from baseline, and being postmenopausal. This is consistent with findings from a previous analysis on dietary supplement use and CIPN in the larger cohort of breast cancer patients enrolled in S0221 [11]. After adjusting for age, race/ethnicity, BMI, smoking status, highest achieved education, and food groups, we observed significant inverse associations between neuropathy and consumption of grains, and marginally significant positive associations with consumption of citrus fruits. We also observed a marginally significant positive association between sensory neuropathy and consumption of sweets.

Few studies have evaluated diet during chemotherapy in relation to neurotoxicity. In a large prospective cohort of breast cancer patients enrolled through Kaiser Permanente Northern California with comprehensive measurements of lifestyle factors, no association between fruit/vegetable intake and clinically worse CIPN was observed [10]. Contrary to this, we did observe a positive association between consumption of citrus fruits and neuropathy symptoms. Due to the cross-sectional design of the study, this observed positive association may be due to the increased consumption of anti-inflammatory foods with high flavones, flavanones, and vitamin C among those attempting to manage their neuropathy. A common myth is that citrus may promote inflammation, although this is mostly triggered by the high fructose content of some citrus fruits [25]. Eating foods that trigger inflammation could increase pain symptoms associated with neuropathy by accelerating the inflammatory process [26]. These foods can vary from person to person, but most commonly include fried foods, sugar-sweetened beverages, red and processed meat, margarine, and refined grains [26]. Due to the design of the FFQ used in this study, we were not able to separate out the potential effects of refined grains alone. However, when we restricted our analyses to sensory neuropathy, we observed a marginally significant positive association with sweets—foods that are high in refined carbohydrates—which may have contributed to an overall inflammatory effect and increased sensory symptomology.

The mechanism by which grain consumption may be associated with decreased neuropathy severity observed in our analysis is unclear. Refined grains tend to have high glycemic load and are associated with increased risk of diabetes and complications, including peripheral neuropathy [27]. Consumption of whole grains, however, is associated with lower risk of diabetes and associated complications due to their antioxidant properties and high levels of fiber [28, 29]. In our study, both refined and whole grains were included in the grain food group, as well as fortified grain products. Although the main dietary sources of vitamin B12 are primarily meat products, fortified grain products can provide a significant amount of vitamin B12 and folic acid [30]. Several vitamin deficiencies, including vitamin E and vitamin B12, have been associated with clinical symptoms similar to that of peripheral neuropathy [31, 32]. Vitamin B12 status has been linked to several neurological disorders, including neuropathy and myelopathy, as well as several brain disorders [33]. Folic acid is another B vitamin associated with neuron growth whose deficiency may increase risk for peripheral neuropathy [34]. In a secondary analysis, we found a significant association between tertile of cold cereal consumption and change in neuropathy symptoms (chi-square *p* = 0.01; data not shown). Those who consumed the highest tertile of cold cereal were less likely to experience an increase in symptoms (*n* = 109, 38.8%). Dietary antioxidants linked to phytochemicals found in the bran and germ fractions of whole grain cereals may help to reduce oxidative stress-mediated neuronal damage, although further research is needed in this area [35, 36].

We compared population characteristics among those included in the analysis and those who did not provide information at baseline regarding neurotoxicity scores and FFQ responses. Those who did not complete the baseline neuropathy assessment were an average of 1.4 years younger. No other significant differences were observed. Those who did not complete baseline FFQs were slightly younger and premenopausal, more obese at baseline, and less likely to be non-Hispanic White, have completed high school, and be married or living as married. Since many of these variables did not modify the association between self-reported diet and neuropathy, it is unlikely that failure to participate created a bias in our results. As with most studies using dietary exposure, the use of self-reported measures may be susceptible to recall bias. Due to the timing of the survey and the cross-sectional design for analysis, the direction of the association is unclear. Diet may have affected CIPN symptoms or CIPN severity may have altered diet. Nausea, vomiting, and loss of appetite are common side effects of chemotherapy that can lead to dramatic changes in diet and weight gain [37]. In this study population, a greater proportion of individuals who reported losing weight during treatment was observed among those with the greatest change in neuropathy scores (10.5%) compared to those with moderate (4.9%) or no change (2.9%). Treatment may increase sensitivity to certain foods, resulting in a deviation from usual diet. This may be true of alcohol consumption, for which the greatest proportion of abstainers was observed among those experiencing severe neuropathy. Further surveys should include an assessment of change in diet due to nausea, dysgeusia, or change in appetite.

There are several, minor limitations in this study. We acknowledge that all participants were part of a large clinical trial and therefore results are not completely generalizable. This was also an exploratory analysis that included multiple hypothesis tests within one model. Although various components of diet were assessed, we did not adjust for multiple comparisons as this analysis was not based on preestablished hypotheses [38]. The purpose of this cross-sectional analysis was to promote additional hypothesis generation for more rigorous analyses. In our analyses, the inclusion of each dietary component was also necessary to control for consumption of that particular food group. We did not observe multicollinearity among food groups (0.35 ≤ *r* ≤ 0.52). Because information on both diet and neuropathy was collected at the same time, there is a lack of temporality. Because alcohol was considered a food in our analyses, there may have been reverse causality from inclusion of both smoking and alcohol consumption. We also made assumptions on missing values, assigning a value of 0 to foods for which participants completed at least 50% of the entire FFQ and a medium serving size when missing. After reviewing the questionnaires, it appeared unlikely that these foods were skipped but that nonresponse was suggestive of lack of consumption.

## Conclusions

While numerous studies on dietary supplements exist, to our knowledge no other studies have examined the associations between a comprehensive diet assessment and CIPN among women with breast cancer. Our analysis was also novel in that we examined various food groups as specific components of diet instead of an aggregate total. We also distinguished between those who experienced a moderate and severe change in neuropathy. By using this approach, we found that citrus fruit and grain consumption may play a role in the neuropathy experience of some women undergoing chemotherapy. This is especially important since there are no existing dietary recommendations for the management of CIPN. Further research utilizing larger, prospective studies is needed to investigate whether there may be certain foods that could worsen or alleviate neuropathy symptoms associated with treatment for breast cancer.

## Abbreviations

BMI: Body mass index
CI: Confidence interval
CIPN: Chemotherapy-induced peripheral neuropathy
DELCaP: Diet, Exercise, Lifestyle, and Cancer Prognosis
FACT/GOG-Ntx: Functional Assessment of Cancer Treatment Gynecologic Oncology Group—Neurotoxicity
FFQ: Food frequency questionnaire
OR: Odds ratio
SWOG: South West Oncology Group
VitaL: Vitamins and Lifestyle
